# Primary Spinal Glioblastoma Mimicking Neuroschistosomiasis: A Case Report

**DOI:** 10.7759/cureus.30248

**Published:** 2022-10-13

**Authors:** Abigail P McCallum, Nicolas K Khattar, Murali K Kolikonda, Sushil Singla, Khaled J Alkhateeb, Alexandra S Schaber, Forest W Arnold, Steven B Lippman, Camilo M Castillo, Brian J Williams

**Affiliations:** 1 Neurosurgery, University of Louisville, Louisville, USA; 2 Neurology, University of Louisville, Louisville, USA; 3 Physical Medicine and Rehabilitation, University of Louisville, Louisville, USA; 4 Pathology and Laboratory Medicine, University of Louisville, Louisville, USA; 5 Infectious Diseases, University of Louisville, Louisville, USA; 6 Psychiatry, University of Louisville, Louisville, USA

**Keywords:** spastic tetraplegia, spinal biopsy, primary spinal tumor, schistosomiasis, spinal glioblastoma

## Abstract

Primary glioblastoma of the spinal cord (sGB) is a rare and challenging diagnosis. In the diagnostic algorithm, reversible causes should be considered while the diagnosis of sGB is under evaluation. We present a case of cervical sGB mimicking neuroschistosomiasis.

A 21-year-old Somali man presented with neck pain, sensory disturbances, and spastic tetraplegia. Cervical spine magnetic resonance imaging with contrast showed a heterogeneously enhancing intramedullary mass spanning from the level of the C1 to T3 vertebrae. Cerebrospinal fluid analysis showed a lymphocytic predominance and elevated protein. Due to the patient’s history of poorly treated schistosomiasis, praziquantel and dexamethasone were initiated while the diagnostic work-up was completed. Three days after the patient was discharged to a rehabilitation facility where he experienced worsened motor function with radiographic progression of the lesion and increased cord edema. The patient underwent a surgical biopsy which confirmed a diagnosis of primary sGB.

sGB is an unusual diagnosis that can masquerade as a non-neoplastic lesion. However, the diagnosis of sGB should be considered in patients with an intramedullary spinal cord lesion who exhibit rapid radiographic and clinical progression.

## Introduction

Primary glioblastoma of the spinal cord (sGB) is a rare and challenging diagnosis, as it accounts for only 1%-3% of all primary spinal cord tumors [1-3]. Despite advances in the diagnosis and treatment of intracranial gliomas, glioblastoma continue to portend a dismal prognosis, with typical survival of 9-11 months from initial diagnosis for sGB with 15-18 months for some intracranial glioblastomas [4-6]. Early diagnosis of sGB is essential to administering adequate treatment. However, the presentation of sGB can mimic other pathologies, including inflammatory, infectious, or non-sGBM neoplastic entities. Herein, we present a case of sGB mimicking spinal neuroschistosomiasis.

## Case presentation

A 21-year-old Somalian male with a recent history of incompletely treated bladder schistosomiasis presented to the emergency department with a three-month history of neck pain associated with paresthesias of both hands and feet, bilateral lower extremity weakness, and gait imbalance causing recurrent falls. The patient’s bladder schistosomiasis was diagnosed six months before the presentation, and he did not complete a full treatment course due to medication non-compliance. Neurological examination revealed spastic tetraplegia, a cape-like sensory loss in the upper extremities, and a stocking-distribution sensory loss to light touch, pain, and temperature.

**Figure 1:**
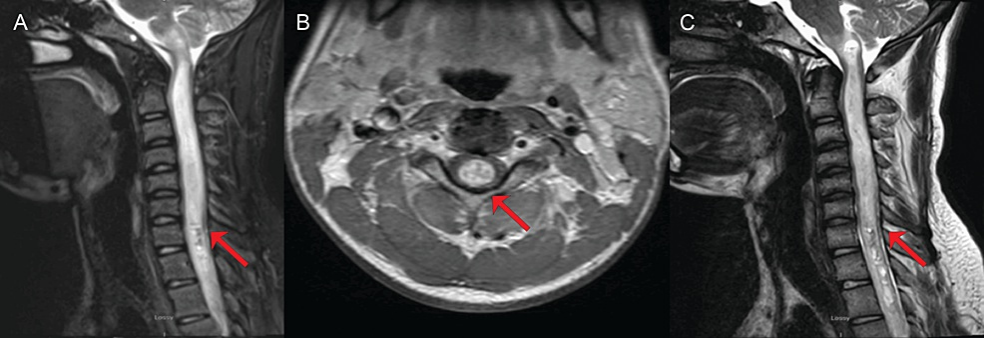
Magnetic resonance imaging of the cervical spine shows the expansile, heterogeneously enhancing intramedullary lesion (A) Sagittal T2-weighted MRI shows a large, hyperintense intramedullary lesion extending from the C1 to T3 vertebral. (B) Axial T2-weighted MRI shows an expansile, hyperintense intramedullary lesion. (C) Follow-up sagittal T2-weighted MRI shows the rostral extension of the lesion into the brainstem with increased surrounding edema. Red arrows show the lesions

Contrast-enhanced magnetic resonance imaging (MRI) of the brain and the cervical spine showed a centrally located intramedullary expansile mass with heterogeneous enhancement. The lesion was associated with adjacent hyperintensity on T2-weighted sequences and punctate foci of gradient echo blooming, superiorly at the level of the obex and inferiorly extending to the T10 vertebral level (Figure 1).

Given the possibility of an infectious etiology, a lumbar puncture was performed. Cerebrospinal fluid (CSF) analysis revealed yellowish fluid, a cell count of 28 nucleated cells with 90% lymphocytic predominance and no red blood cells, normal glucose, and elevated protein with a level >300 mg/dL. We made a putative diagnosis of neuroschistosomiasis, and the patient was started on praziquantel and dexamethasone. The patient’s symptoms improved, and he was discharged to an acute inpatient rehabilitation center. 

**Figure 2:**
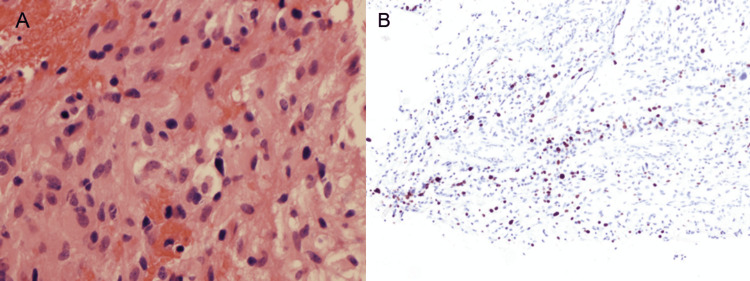
Histopathological samples from the spinal cord biopsy (A) Hematoxylin and eosin-stained photomicrograph (40 x magnification) shows a highly cellular tumor with areas of necrosis, significant pleomorphism, nuclear atypia, and increased mitotic activity (B) MIB-1 immunohistochemical staining shows a high proliferative index (10x magnification)

Three days later, the patient’s upper and lower extremity motor strength significantly deteriorated. Repeat cervical spine MRI showed significant rostral extension of the lesion into the medulla with increased fluid attenuated inversion recovery (FLAIR) hyperintensity. Repeat lumbar puncture showed an unchanged CSF analysis. The initial diagnosis of neuroschistosomiasis was questioned at this time, and a spinal cord biopsy was performed. Histopathological analysis of the lesional tissue was consistent with sGB, including findings of pleomorphism, high cellularity, vascular proliferation, and central necrosis. The tumor cell populations were bimodal, such that some cells had small cytoplasms with hyperchromatic nuclei, whereas others had large cytoplasms with pleomorphic nuclei and gemistocyte features (Figure 2).

Immunohistochemistry showed MIB-1 and p53 labeling indices of 17.1% and 65%, respectively. Despite the best medical therapy, the patient’s neurological symptoms progressed and involved his respiratory muscles, thereby causing cardiorespiratory failure within 48 hours of the biopsy.

## Discussion

sGB represents only 1%-3% of all primary spinal tumors [1-3]. This contrasts sharply with intracranial glioblastoma, which represents 50% of all primary malignant brain tumors [7]. The median age at presentation for sGB is 22-35 years, with less than 6% of patients older than 60 years [5]. In the early 1980s, the prognosis of both spinal and intracranial glioblastoma was thought to be equivalent. While the survival of patients with intracranial glioblastoma has gradually improved over time (median duration 15-22 months), patients with sGB continue to have a relatively low survival of 9-11 months [4-6]. Treatment strategies for sGB remain controversial with variable outcomes. Radical surgical excision showed a modest survival benefit in some series but not in others [5,8,9]. For sGBs involving the lower thoracic cord and below, cordectomy is a viable surgical option. To date, cordectomy has been the only intervention reported to considerably improve survival, with durations of 14-144 months [8,10-12]. However, cordectomy was not feasible for our patient’s cervical sGB.

It remains challenging to establish a diagnosis of sGB. CSF and serological testing are useful adjuncts to distinguish among the various etiologies of an intramedullary lesion [13]. The differential diagnosis for an intramedullary lesion includes infection, demyelination, inflammation, and neoplasia. A progressive neurological decline in the setting of medical treatment failure is an indication of spinal cord biopsy [13]. Some entities on the differential diagnosis of a cervical intramedullary lesion, such as neuroschistosomiasis, are reversible and can be medically treated [14,15]. *Schistosoma haematobium* and *S. mansoni* are the most common parasitic infections of the spinal cord, and they can cause acute and chronic spinal complications. Schistosomaiasis can be curatively treated with a course of praziquantel. Spinal schistosomiasis usually affects the lower thoracic region or cauda equina [15]. Given the morbidity associated with spinal cord biopsies, initial treatment with antiparasitic agents is a reasonable first step in the management algorithm for intramedullary spinal cord lesions, which includes a therapeutic trial for any reversible or medically treatable causes [16]. Since the diagnostic testing to confirm neuroschistosomiasis is difficult and time-consuming, initiating early empiric treatment is indicated.

For cervical sGB, no therapeutic intervention has shown any survival advantage to date, and early treatment is not as essential as for an infectious etiology. Currently, sGB remains an aggressive pathology with a dismal prognosis. Clinicians managing a patient with an intramedullary spinal cord lesion should have an appropriate level of suspicion for this neoplastic entity. The risk-to-benefit profile of spinal cord biopsy must be carefully weighed, and surgery should be performed on carefully selected patients after an appropriate evaluation.

## Conclusions

sGB is a very rare diagnosis that can mimic non-neoplastic processes. One should consider the diagnosis of sGB in patients with an intramedullary spinal cord lesion who develop precipitous radiographic and clinical progression. 

## References

[1] Asano N, Kitamura K, Seo Y, Mukai K, Soga T, Hondo H, Matsumoto K (1990). Spinal cord glioblastoma multiforme with intracranial dissemination--case report. Neurol Med Chir (Tokyo).

[2] Medhkour A, Chan M (2005). Extremely rare glioblastoma multiforme of the conus medullaris with holocord and brain stem metastases, leading to cranial nerve deficit and respiratory failure: a case report and review of the literature. Surg Neurol.

[3] Morais N, Mascarenhas L, Soares-Fernandes JP, Silva A, Magalhães Z, Costa JA (2013). Primary spinal glioblastoma: A case report and review of the literature. Oncol Lett.

[4] Santi M, Mena H, Wong K, Koeller K, Olsen C, Rushing EJ (2003). Spinal cord malignant astrocytomas. Clinicopathologic features in 36 cases. Cancer.

[5] Adams H, Avendaño J, Raza SM, Gokaslan ZL, Jallo GI, Quiñones-Hinojosa A (2012). Prognostic factors and survival in primary malignant astrocytomas of the spinal cord: A population-based analysis from 1973 to 2007. Spine (Phila Pa 1976).

[6] Beyer S, von Bueren AO, Klautke G, Guckenberger M, Kortmann RD, Pietschmann S, Müller K (2016). A systematic review on the characteristics, treatments and outcomes of the patients with primary spinal glioblastomas or gliosarcomas reported in literature until March 2015. PLoS One.

[7] Kohler BA, Ward E, McCarthy BJ (2011). Annual report to the nation on the status of cancer, 1975-2007, featuring tumors of the brain and other nervous system. J Natl Cancer Inst.

[8] Curran WJ Jr, Scott CB, Horton J (1993). Recursive partitioning analysis of prognostic factors in three Radiation Therapy Oncology Group malignant glioma trials. J Natl Cancer Inst.

[9] McGirt MJ, Blessing R, Alexander MJ (2006). Risk of cerebral vasopasm after subarachnoid hemorrhage reduced by statin therapy: A multivariate analysis of an institutional experience. J Neurosurg.

[10] Marchan EM, Sekula RF Jr, Jannetta PJ, Quigley MR (2007). Long-term survival enhanced by cordectomy in a patient with a spinal glioblastoma multiforme and paraplegia. Case report. J Neurosurg Spine.

[11] König SA, Roediger T, Spetzger U (2012). Treatment of recurrent primary spinal glioblastoma multiforme--case report. J Neurol Surg A Cent Eur Neurosurg.

[12] Viljoen S, Hitchon PW, Ahmed R, Kirby PA (2014). Cordectomy for intramedullary spinal cord glioblastoma with a 12-year survival. Surg Neurol Int.

[13] Cohen-Gadol AA, Zikel OM, Miller GM, Aksamit AJ, Scheithauer BW, Krauss WE (2003). Spinal cord biopsy: a review of 38 cases. Neurosurgery.

[14] Alsomaili M, Abulaban AA (2016). Spinal cord schistosomiasis: Two different outcomes. Case Rep Neurol.

[15] Ashour AM, Elserry TH, Nosser MS (2018). Spinal schistosomiasis: Cases in Egyptian population. J Craniovertebr Junction Spine.

[16] Dormegny L, Chibbaro S, Ganau M, Santin M, Kremer L, Proust F (2018). Biopsying a spinal cord lesion: A diagnostic dilemma. Case report and review of literature. Neurochirurgie.

